# Mutation of the Carboxy-Terminal Processing Protease in *Acinetobacter baumannii* Affects Motility, Leads to Loss of Membrane Integrity, and Reduces Virulence

**DOI:** 10.3390/pathogens9050322

**Published:** 2020-04-26

**Authors:** Rakesh Roy, Ren-In You, Ming-Der Lin, Nien-Tsung Lin

**Affiliations:** 1 Institute of Medical Sciences, Tzu Chi University, Hualien 97004, Taiwan; rakeshroy1803@gmail.com; 2 Institute of Medical Biotechnology, Tzu Chi University, Hualien 97004, Taiwan; yri100@gms.tcu.edu.tw; 3 Department of Molecular Biology and Human Genetics, Tzu Chi University, Hualien 97004, Taiwan; mingder@gms.tcu.edu.tw; 4 Department of Microbiology, School of Medicine, Tzu Chi University, Hualien 97004, Taiwan

**Keywords:** *Acinetobacter baumannii*, Tn*10* transposon, carboxy-terminal processing protease (Ctp), motility, mucoidy, adhesion, invasion

## Abstract

Motility plays an essential role in the host–parasite relationship of pathogenic bacteria, and is often associated with virulence. While many pathogenic bacteria use flagella for locomotion, *Acinetobacter baumannii* strains do not have flagella, but have other features that aid in their motility. To study the genes involved in motility, transposon mutagenesis was performed to construct *A. baumannii* mutant strains. Mutant strain MR14 was found to have reduced motility, compared to wild-type ATCC 17978. NCBI BLAST analysis revealed that the Tn*10* transposon in the MR14 genome is integrated into the gene that encodes for carboxy-terminal processing protease (Ctp). Additionally, MR14 exhibits a mucoidy, sticky phenotype as the result of increased extracellular DNA (eDNA) caused by bacterial autolysis. Transmission and scanning electron microscopy revealed cytoplasmic content leaving the cell and multiple cell membrane depressions, respectively. MR14 showed higher sensitivity to environmental stressors. Mutation of the *ctp* gene reduced invasion and adhesion of *A. baumannii* to airway epithelial cells, potentially due to increased hydrophobicity. In the zebrafish model of infection, MR14 increased the survival rate by 40% compared to the wild-type. Taken together, the *ctp* gene in *A. baumannii* has a pivotal role in maintaining membrane integrity, adaptation to environmental stress, and controlling virulence.

## 1. Introduction

*Acinetobacter baumannii* is a multi-drug resistant (MDR), nosocomial human pathogen causing serious infections among critically ill individuals worldwide. This microorganism is usually involved in various hospital and community-associated diseases, with MDR rates being higher among nosocomial isolates than strains causing community-associated infections [1]. *A. baumannii* can cause diverse infections, including bacteremia, meningitis, urinary tract infections, wound infections, septic shock, ventilator-associated pneumonia, and systemic infections leading to multi-organ failure and death among critically ill and immunocompromised individuals [2,3]. Currently, *A. baumannii* is resistant to almost all presently available antibiotics, and is termed a “red-alert” pathogen [4]. Treatment of infections caused by this pathogen is challenging, and there is an urgent need for the development of novel antimicrobial therapeutic strategies.

Flagella are important locomotive organelles used by various pathogenic bacteria to cause diseases in humans [5]. Genome sequence analysis of *A. baumannii* has revealed the absence of genes required for flagella biosynthesis, necessary for swarming motility [6]. Hence, they are described as non-motile. However, the genome of *A. baumannii* revealed the presence of genes required for type IV pili assembly [7]. In semi-solid media, *A. baumannii* still exhibits flagellum-independent motility, such as twitching motility and surface-associated motility [8,9,10]. These motility phenotypes of *A. baumannii* are regulated by numerous mechanisms that include the activity, for example, of type IV pili [6,11], light-dependent type I pilus [12], extracellular stress and lipopolysaccharides [13], several environmental factors [14], and the quorum sensing network [6]. The motility of bacteria is regulated by a complex signal transduction system [15]; in *Pseudomonas aeruginosa* and *Escherichia coli*, around 44 and 60 genes, respectively, have been identified that are associated with motility [16,17]. In *A. baumannii*, type IV pili are found to play an essential role in twitching motility and natural transformation [10], contribute to adherence to host cells [18], but it does not regulate surface-associated motility [11]. Other than type IV pilus, one researcher identified photo-regulated type I pilus that is associated with cell surface motility, adhesion, biofilm, and virulence [12]. The number of genes responsible for controlling the motility of *A. baumannii* is less explored, and little is known about the link between motility and virulence. Several studies have reported the importance of motility for the colonization of bacteria and the initiation of infections [19,20,21]. Motility among *A. baumannii* from blood isolates is a common phenotype compared to respiratory isolates [22]. *A. baumannii* with reduced motility has been shown to have decreased virulence in a caterpillar model of infection [23]. Hence, considering the importance of motility in pathogenesis, and the need for a novel approach against MDR *A. baumannii*, therapeutic strategies targeting motility could be a good option to control this pathogen. The aim of this study was to identify genes associated with motility in *A. baumannii*, and to investigate their association with virulence.

## 2. Results

### 2.1. Carboxy-Terminal Processing Protease (Ctp) Involvement in *A. baumannii* Motility

**Figure 1:**
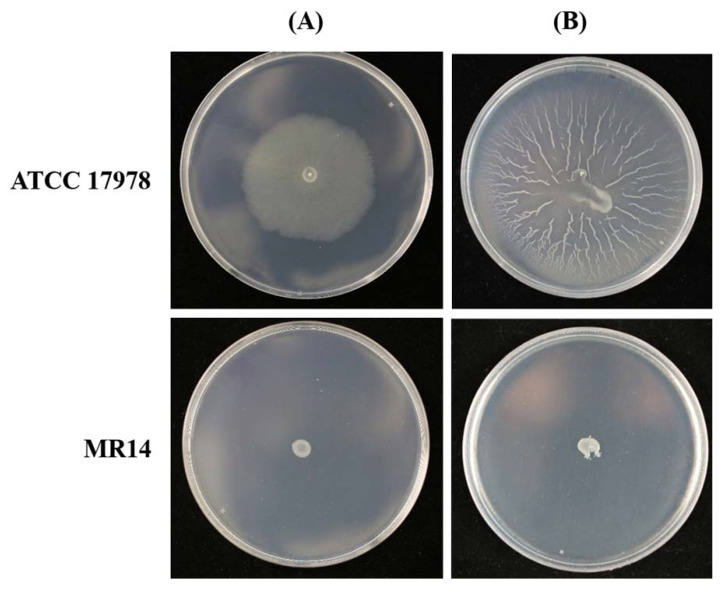
The surface-associated motility (**A**) and twitching motility (**B**) defect of mutant MR14. Phenotypes of parental strain *Acinetobacter baumannii* ATCC 17978 and the transposon insertion mutant MR14 on 0.5% agar motility plates incubated for 16 h at 37 °C. (**A**) A 1 µL drop of each strain was placed on the surface of semisolid agar plate, and (**B**) stab-inoculated into the interphase between the bottom of the agar layer and the polystyrene Petri dish.

The mini-Tn*10* transposon mutagenesis method allowed us to obtain a library of approximately 1500 *A. baumannii* ATCC 17978 mutants. A motility assay was performed to screen for mutant(s) with impaired motility. Mutant MR14 significantly lost motile ability when compared to the parental strain, ATCC17978, as apparent by both twitching and surface-associated assays (Figure 1).

**Figure 2:**
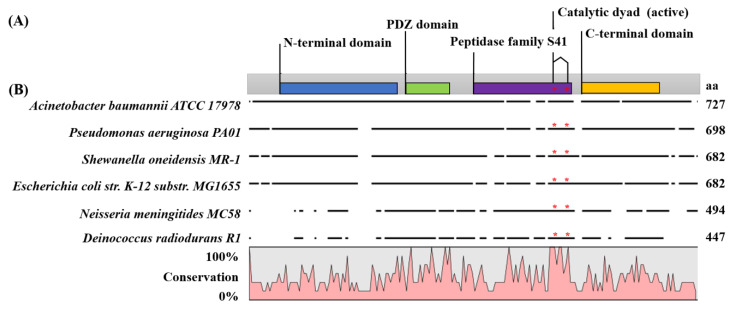
Domain structure and multiple sequence alignment of *A. baumannii* Ctp and S41 family members from other bacteria. (**A**) Four domains were identified by InterPro-EMBL-EBI search of *A. baumannii* Ctp protein sequence. The N-terminal domain is located from amino acids 56–261 (blue box), a PDZ domain from amino acids 276–352 (green box), an S41 peptidase domain from amino acids 395–568 (purple box), and a C-terminal domain from amino acids 574–722 (yellow box). The S41 protease catalytic dyad (consisting of aa S503 and K528) in the S41 domain is indicated by red asterisk. (**B**) Multiple sequence alignment of S41 family members from different Gram-negative bacteria, including *A. baumannii* (ABO10946.2), *Pseudomonas aeruginosa* (NP_251947.1), *Shewanella oneidensis* (NP_718187.1), *Escherichia coli* (NP_416344.1), *Neisseria meningitides* (NP_ 274351.1), and *Deinococcus radiodurans* (NP_295274.1), was performed using CLC Genomics Workbench 20.0.2. A line plot was generated to display similar/identical residues. The color scale in the line plot varies from 0% to 100%, representing a low and high conservation of amino acid sequences, respectively.

To identify the disrupted gene, we performed inverse PCR to amplify the flanking region of the Tn*10* insert. After sequencing the inverse PCR product, we found that the Tn10 insertion was within A1S_0493, at a position corresponding to amino acid (aa) 378 of the 728 aa protein. The aa sequence of A1S_0493 encoded a protein that exhibited significant homology to the tail specific protease (Tsp) of *P. aeruginosa* (35% identity and 55% similarity), *E. coli* str. K-12 substr. MG1655 (33% identity and 51% similarity), the periplasmic C-terminal processing protease (Prc) of *Shewanella oneidensis* MR-1 (35% identity and 52% similarity), and the carboxy-terminal processing protease (Ctp) of *Neisseria meningitidis* MC58 (32% identity and 48% similarity) and *Deinococcus radiodurans* R1 (29% identity and 44% similarity) (Figure 2). A BLAST search showed the *A. baumannii* A1S_0493 encoded protein sequence demonstrated PDZ domains located between aa 276 and 352, while an S41 peptidase domain was located between residues 395 and 568, and a catalytic dyad (corresponding to the S41A subfamily of the MEROPS database) formed by serine and lysine at aa 503 and 528 (Figure 2). The presence of these domains suggests A1S_0493 to be annotated as Ctp, a new member of serine proteases involved in the C-terminal processing proteins. In addition, a 28 aa N-terminal signal peptide was predicted by signalP-5.0, which indicates a possible translocation across the cytoplasmic membrane by the Sec-pathway.

### 2.2. Complementation of MR14 with *Ctp* Gene Can Restore Twitching and Surface-Associated Motility

**Figure 3:**
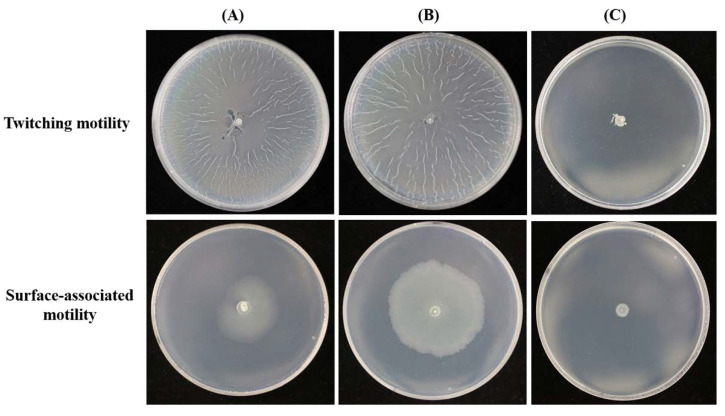
Complementation of the Ctp mutant restores motility. (**A**) The plasmid-borne wild-type *ctp* gene (pABCL_11b-0493) introduction into MR14 strain restores the twitching and surface-associated motility phenotype. (**B**,**C**) The twitching and the surface-associated motility phenotype of ATCC 17978 and MR14 strains, respectively, carrying the shuttle vector, pABCL_11b, is similar to that of their respective strains without shuttle vector (Figure 1).

To confirm that Tn*10* inserted in the *ctp* gene is responsible for the loss of motility, the chromosomal DNA region containing the A1S_0493 locus was amplified by PCR. The product was cloned into pABCL_llb and transformed into MR14. One representative transformant, MR14C, was characterized by restoring motility phenotype (Figure 3). As detailed in the following section, MR14C complemented all of the phenotype properties of the Ctp mutant, that include mucoidy and sticky phenotype, growth defect post 18–24 h of culture, bacterial morphology, and cell surface hydrophobicity, indicating that the disruption of *ctp* was responsible for the mutant phenotype.

### 2.3. MR14 Showed Mucoidy and Sticky Phenotype

**Figure 4:**
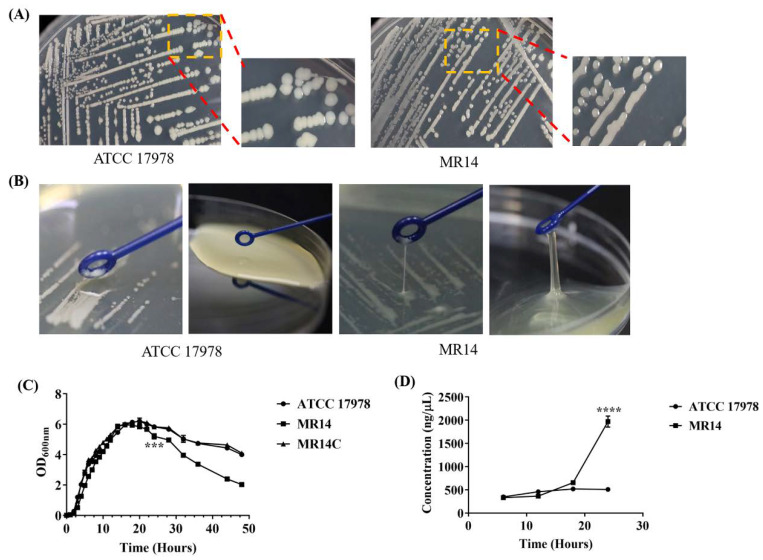
Colony appearance and growth curve of culture ATCC 17978 and Ctp mutant MR14. (**A**) The wild-type ATCC 17978 formed non-mucoid colonies on LB agar (left), and the Ctp mutant MR14 formed mucoid, shiny colonies (right). (**B**) When touched with an inoculation loop, the MR14 strain formed mucoidy, sticky strands on the LB plate after five days of incubation at 4 ℃, and on LB media after incubation for 18 h (right) in contrast to ATCC 17978 (left). (**C**) Growth curve analysis shows a reduced OD_600_ value of MR14 after 18 h of culture compared to ATCC 17978. (**D**) Extracellular DNA (eDNA) quantification over the temporal scale of growth. The error bars represent standard error of the mean obtained from three independent experiments. *** *p* < 0.001, **** *p* < 0.0001.

Unlike dry, non-mucoid wild-type ATCC 17978, we observed that MR14 forms shiny, mucoid colonies (Figure 4A). MR14 also tends to become sticky and slimy after 3–5 days on agar plates and 16–18 h of culture in LB (Figure 4B). A previous study reported stickiness and viscous growth of bacteria in liquid to be due to extracellular deoxypentose nucleic acid (DNA) slime [24]; also, eDNA is said to be a part of slimy matrix produced by bacteria [25]. We predicted that the mucoid and sticky nature of MR14 is due to the accumulation of extracellular DNA (eDNA), as we also observed that during the mucoidy state, MR14 showed a significantly reduced OD_600_ value compared to the wild-type (Figure 4C). To confirm this, we added DNase I (2U) to MR14 while it was mucoid and further incubated it at 37 °C for 2 h. As predicted, after the addition of DNase I, the MR14 could no longer form the mucoidy or sticky phenotype. Furthermore, we performed eDNA purification over the temporal scale of growth and observed that during the mucoidy state, MR14 had significantly increased eDNA compared to the wild-type ATCC 17978 (*p* < 0.0001) (Figure 4D).

### 2.4. The *Ctp* Gene is Essential for Maintaining Membrane Integrity

**Figure 5:**
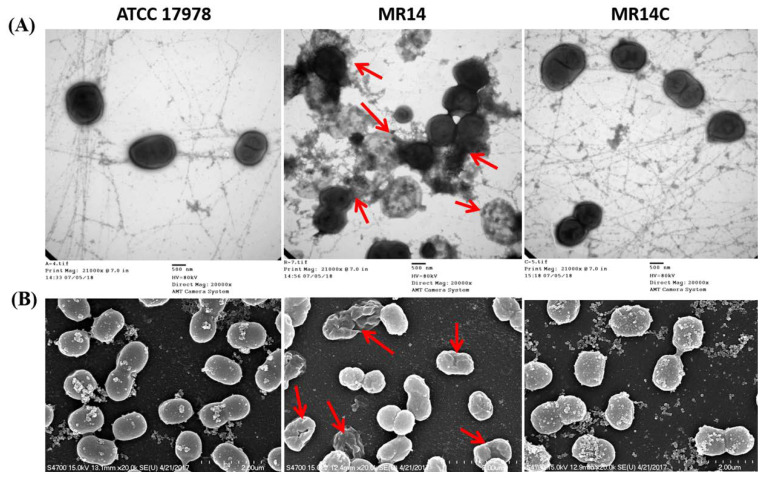
Cell morphology visualization. (**A**) Cell morphology visualization by TEM. Columns from left to right are wild-type ATCC 17978, MR14, and MR14C. The red arrows show damaged cell walls. (**B**) Cell morphology visualization by SEM. Columns from left to right are wild-type ATCC 17978, MR14, and MR14C. The red arrows indicate indentions and depressions. Scale bar = 500 nm.

The *A. baumannii* strains were visualized by TEM and SEM to analyze morphological and structural changes. Under TEM, the wild-type and the complementation strains showed a normal shape with an undamaged membrane structure. Both the inner and outer membrane was found to be intact. In contrast, the MR14 showed a damaged cell wall structure, causing cytoplasmic content to be released from the cells (Figure 5A). The results of SEM were consistent with the TEM results. Both the wild-type and complementation strains showed a smooth cell surface with no detectable damage. The MR14 showed a damaged cell wall structure, with multiple depression and indentations (Figure 5B). This suggests that disruption of *ctp* induced cell wall disruption, leading to a loss of membrane integrity. This result implies that the eDNA released, as mentioned above, is the result of membrane integrity loss and membrane damage caused by a *ctp* gene mutation.

### 2.5. The *Ctp* Gene Mutation Induces Cell Lysis

**Figure 6:**
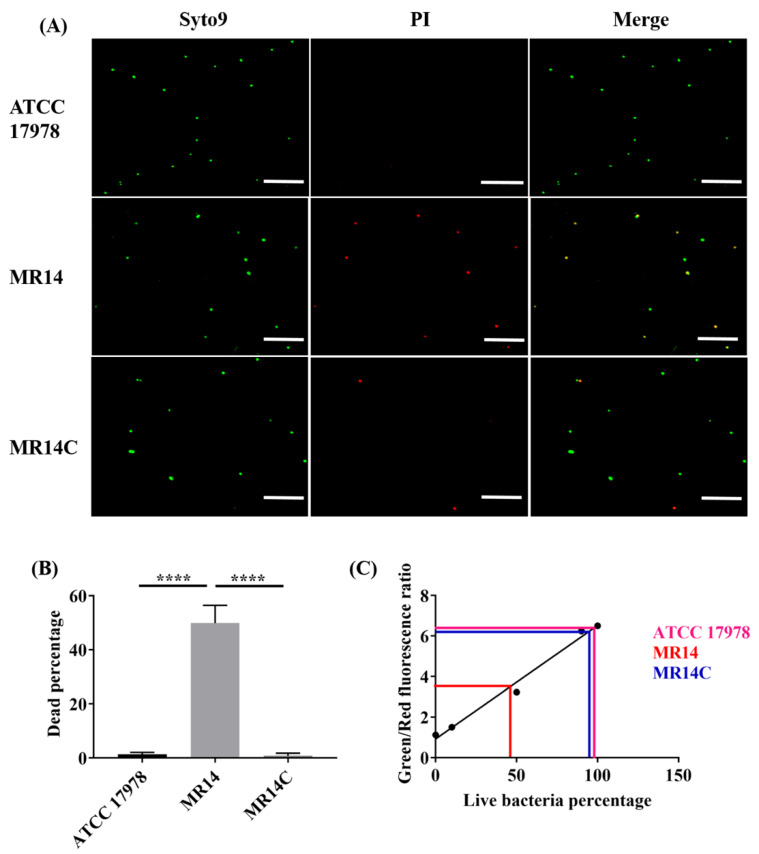
The effect of the *ctp* gene mutation on *A. baumannii* viability. (**A**) Confocal scanning laser microscopy images of fluorescence-stained ATCC 17978, MR14, and MR14C using a live/dead BacLight^TM^ bacterial viability kit; scale bar = 20 µm; (**B**) number of dead cells obtained from fluorescence microscopy using live/dead staining; (**C**) microtiter fluorescent method for the quantification of live and dead bacterial percentages, a linear relationship between the percentage of live cells and the green/red fluorescence ratio from the SYTO9/propidium iodide (PI) viability assay. All experiments were performed in triplicate. **** *p* < 0.0001.

The TEM and SEM analysis of the mutant strain showed a loss of membrane integrity (Figure 5). Compared to the wild-type, MR14 has a reduced OD_600_ value after 16–18 h of culture. We anticipated that this was due to the autolysis of MR14 caused by loss of membrane integrity. To confirm this hypothesis, we checked the viability of *A. baumannii* strains by live/dead double staining, followed by visualization of stained cells using confocal microscopy (Figure 6A). The percentage of red-stained cells, which indicated the percentage of dead cells, was 1.3% and 0.8% for the wild-type and complementation strains, respectively, whereas the proportion of red-stained cells for the mutant strain was 49.8% (Figure 6B). The percentage of live bacteria obtained from the fluorescence microplate method was 97.9% and 94.7% for wild-type and complementation strains, respectively, while the mutant strain was found to have 46.9% live bacteria (Figure 6C). These results suggested that the *ctp* gene mutation in *A. baumannii* leads to bacterial death by altering membrane integrity.

### 2.6. The *Ctp* Gene Contributes to Tolerance against External Stresses

**Figure 7:**
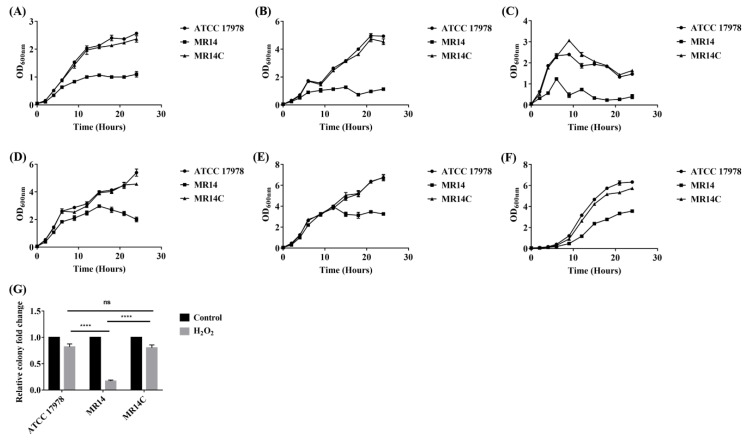
The effect of the Ctp mutation on the stress tolerance of *A. baumannii*. (**A**) 3% NaCl; (**B**) 3% alcohol; (**C**) 1% Triton X-100; (**D**) pH 5.5; (**E**) pH 8.5; (**F**) 20 °C; (**G**) 20 mM H_2_O_2_. The bacterial growth under different stress conditions was monitored by measuring OD_600_. For stress under hydrogen peroxide, the relative cell survival was calculated relative to the untreated control. The results are expressed as means ± SE. *n* = 3, **** *p* < 0.0001, ns = not significant.

The ability of bacteria to tolerate environmental stress is crucial for their virulence. Bacteria that can persist under various environmental stress conditions are considered a serious threat to human health worldwide. The growth of *A. baumannii* strains was studied under various stress conditions to determine the effect of the *ctp* gene on stress tolerance. Compared to wild-type and complementation strains, the mutant strain showed reduced growth upon exposure to different stresses, such as oxidative stress, temperature, pH, and osmotic pressure (Figure 7). This result implies that the *ctp* gene contributes to the tolerance of external stresses, especially NaCl, alcohol, Triton X-100, and H_2_O_2_.

### 2.7. The *Ctp* Gene Contributes to Virulence in *A. baumannii*

**Figure 8:**
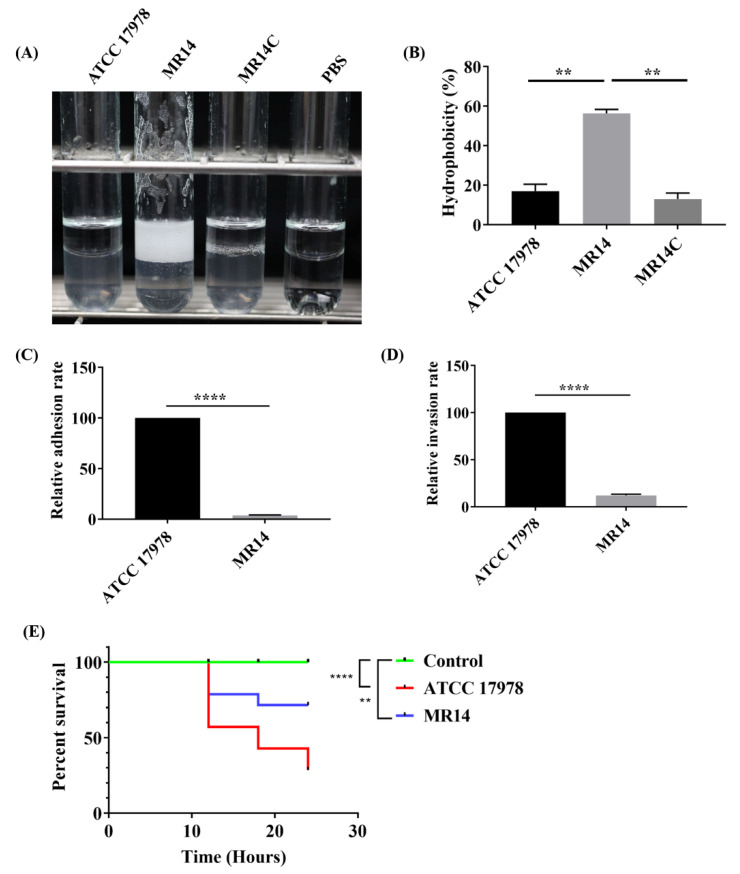
Ctp is required for virulence. (**A**) Cell surface hydrophobicity analysis using xylene, left to right is ATCC 17978, MR14, MR14C, and control; (**B**) cell surface hydrophobicity in terms of percentage. (**C**) Adhesion assay. (**D**) Invasion assay. The adhesion and invasion of the wild-type strain were set at 100%, and MR14 strain data are reported relative to these values. Data are from three independent experiments. (**E**) Two groups, each consisting of 14 zebrafish, were infected with 10^6^ colony forming units (CFU) of wild-type or MR14 via cloaca injection. Zebrafish survival was monitored every 6 h for 24 h. The control group represents zebrafish injected with PBS. **** *p* < 0.0001, ** *p* < 0.01.

Bacterial cell surface hydrophobicity is related to virulence-associated processes, and plays an essential role in the adhesion of bacteria to a wide variety of surfaces [26,27]. The deletion of *prc* in *E. coli* is reported to change the outer membrane protein profile [28]. One of the functions of outer membrane proteins is to maintain outer membrane integrity, and our result shows membrane damage of MR14 strain (Figure 6). Hydrophobicity of the cell surface is also reported to be influenced by outer membrane and surface proteins and LPS [29]. Additionally, when we observed the cell pellet pattern after centrifugation, we found that MR14 had relatively longer cell pellet patterns, as described previously [30], and the pellet forms a clump that cannot be easily resuspended in either ddH_2_O, PBS, or LB medium (data not shown). We hypothesized this phenotype to be due to a change in cell surface hydrophobicity. The cell surface hydrophobicity of the MR14 strain was found to increase significantly (Figure 8A,B). The results imply that Ctp changes the cell surface properties, making it hydrophilic for easy attachment to biotic surfaces.

Adherence of bacteria is the initial step in their colonization, and thus an important virulence factor in infection and disease progression. Additionally, the invasion of bacteria into host cells helps them escape humoral immunity and allows them to penetrate deep into the tissues [31]. In this study, we assessed the adherence and invasion of *A. baumannii* strains in human lung epithelial cells (A549). Compared to the wild-type strain, ATCC 17978, MR14 exhibited reduced adherence and invasion properties (Figure 8C,D). The increased hydrophobicity and decreased adhesion and invasion are in accordance with a previous study [32]. This result implies that the *ctp* gene mutation impairs both the adhesion and invasion of *A. baumannii* to A549 cells. Since *ctp* is a stress response gene, it is hypothesized to play an important role for this pathogen to survive stress-inducing conditions during invasion and adhesion of host cells. Furthermore, several reports have demonstrated the role of the *ctp* gene in virulence [33,34,35]. The zebrafish infection model was selected to assay the role of *ctp* in the pathogenesis of *A. baumannii* infection. Zebrafish injected with 1 × 10^6^ colony forming units (CFU) were found to have an LD_50_ at 24 h. We observed a significantly higher survival rate in zebrafish infected with the MR14 strain (70%) compared to those infected with the wild-type strain (30%) (Figure 8E).

## 3. Discussion

This study was aimed at the identification of genes responsible for the motility of *A. baumannii* on low percentage of agar. Tn*10* transposon mutagenesis of the *ctp* gene was found to contribute to significantly reduced motility. Our next aim was the functional characterization of this gene, and to assess its relationship with virulence. The *ctp* gene has been previously identified and characterized in different Gram-negative and Gram-positive bacterial species, such as *P. aeruginosa, Burkholderia mallei*, *Borrelia burgdorferi, Bacillus subtilis*, and *Staphylococcus aureus* [33,36,37,38,39]. The *ctp* gene mutants have shown pleiotropic phenotypes among different bacteria and that could be due to the diversity in sequence [40]. Due to variable phenotypes and diversity in sequence shown by this gene, it can be inferred that this gene has unique functions in each bacterial species. To our knowledge, for the first time, we have identified and characterized the *ctp* gene in *A. baumannii* with some unique characteristics that have not been previously reported. These characteristics include reduced motility, mucoid and sticky phenotypes, hydrophobicity, and reduced virulence.

The experimental evidence from motility assays performed on 0.5% agar demonstrates that the Ctp mutant strain is defective in terms of motility phenotype. The exact mechanism behind this significantly reduced motility remains unclear. The Ctp mutant also becomes mucoidy, which we found is due to the accumulation of eDNA caused by bacterial autolysis. Formation of the mucoidy phenotype might cause the bacterial cells to adhere to one another, resulting in the arrest of motility. The bacterial autolysis is attributed to a loss of membrane integrity [41,42]. Compromised membrane integrity is found to be associated with reduced motility phenotype [43]. Our TEM and SEM analyses clearly show the loss of membrane integrity by MR14. Further, we confirmed the compromised membrane integrity of MR14 via cell viability assays using confocal microscopy and microtiter formats. A previous study reported a broad impact on the ABC-binding-cassette transport system and thus nutrient uptake efficiency due to Ctp mutation in *Rhizobium leguminosarum* [44], resulting in a slow growth rate. On the LB agar plate, we observed a reduction in colony size for MR14 compared to the wild-type strain. The reduced colony size is attributed to the slow growth rate [45,46]. Based on our observation, the reduced colony size of MR14 could be due to impaired growth rate caused by growth arrest or autolysis, however, further research is required to verify the underlying factors. One possible reason is the defect in peptidoglycan metabolism mechanism. Penicillin-binding proteins are involved in the synthesis of peptidoglycan, a major component of bacterial cell walls. In *Salmonella* and *B. subtilis*, penicillin-binding proteins are reported to be responsible for cell division through septum formation [47,48]. Prc has been identified to process its substrate, penicillin-binding protein 3, a peptidoglycan transpeptidase that is required for cell division [49]. Prc is also found to process another peptidoglycan-associated substrate, MepS, that is required for cleavage of cross-link between glycan chains of peptidoglycan [50]. Taken together, we hypothesize that the cell lysis and reduced colony size of MR14 strain are due to a lack of processing of protein substrates associated with peptidoglycan metabolism. Growth arrest or lysis of bacterial cells is also reported to be due to an imbalance in the biosynthesis of peptidoglycan caused by inhibition of the penicillin-binding proteins [51]. The change in the phenotype of Ctp mutant is supposed to be the result of substrate dysregulation due to loss of *ctp* gene function. The substrate of Ctp in *A. baumannii* remains unknown and needs further experiments. Tsp determines its substrate based on the presence of apolar residues and a free α-carboxylate at C-terminus, and cleave the peptide bond with Ala, Ser, or Val at the P1 position and these same residues with Met, Tyr, or Trp at the P1’ position [52]. Proteins with nonpolar C termini are demonstrated to be the target of Tsp [53], and based on this, one of the reports predict most of the potential target of Ctp to be putative outer membrane proteins and transporter components using 2D gel electrophoresis [44]. The CtpA in *Borrelia burgdorferi* targets outer membrane proteins that include P13, BB0323, BBA01, and OspC [38,54,55]. The P13 and BBA01 are outer membrane porins and have channel-forming activity whereas, BB0323 and OspC, are virulence-associated lipoproteins required for cell fission, to persist in mice and to adhere and invade tick salivary glands, respectively [54,56,57]. Phenotypes in *B. burgdorferi,* like slower growth rate of *ctpA* mutant and the role of substrate of *ctpA* in adhesion and invasion to its host cell, are consistent with our results. Hence, outer membrane lipoproteins and porins could be predicted as the possible targets of Ctp of *A. baumannii*. Recent research on the extraintestinal pathogenic *E. coli* shows that Prc is associated with bacterial motility, envelope integrity, and virulence [58]. All phenotypes of *prc* deleted mutant of *E. coli* are similar to the phenotypes of MR14 strain in our study. Though they demonstrated that the reduced motility is associated with the downregulation of transcriptional regulator of flagellum biogenesis, and gene for flagella is absent in *A. baumannii*, we hypothesized that all these phenotypes are the outcome of altered protein expression in the membrane fraction of Ctp mutant for e.g., altered peptidoglycan synthesis leading to loss of membrane integrity and finally this compromised membrane results in the attenuation or downregulation of other genes or proteins responsible for motility, interaction with the host, and virulence. Further proteomics study is required to find the specific substrate of the Ctp of *A. baumannii* and to correlate the downstream effect of Ctp with the altered phenotype.

Several reports have demonstrated that Ctp is an important factor for stress tolerance, with stressors including heat, osmotic pressure, desiccation, and detergents [49,59]. Ctp also maintains PSII activity in response to environmental stress in the cyanobacterium *Synechocystis* [60]. The periplasmic protease Prc in *Salmonella typhimurium* has been shown to contribute to survival under macrophage-induced acidic environmental stress [61]. The Ctp in *E. coli* is reported to degrade proteins translated from damaged mRNA [62]. It is apparent that the *ctp* gene plays an important role in response to stress conditions. Since *A. baumannii* is ubiquitous, it has to face various stress conditions to be able to successfully infect its hosts. However, the association of Ctp and stress-related proteins in *A. baumannii* remains to be determined; our results show that the mutation of *ctp* makes *A. baumannii* hypersensitized to environmental stresses.

*A. baumannii* has been reported to use OmpA and appendages like pili, fimbrial-like structures for the adhesion and invasion of host cells [31,63,64]. Appendages like pili and fimbriae contribute to the motility, while OmpA plays an important role in maintaining membrane integrity [65]. From the reduced motility phenotype and loss of membrane integrity that we obtained in our results, we hypothesize that the Ctp mutation affects the expression and/or function of proteins like OmpA, pili, and fimbriae, which in turn affect the motility, adhesion, and invasion characteristics of the pathogen. Furthermore, the loss of membrane integrity and hence increased sensitivity to stress is deduced to weaken its ability to adhere and invade epithelial cells. Based on our results (Figure 8A,B), we suggest that the *ctp* gene mutation leads to increased bacterial surface hydrophobicity. Previously, hydrophobic *A. baumannii* was demonstrated to have reduced adherence to lung epithelial cells compared to the hydrophilic *A. baumannii* strains [32], which is in accordance with our results. Our results on virulence using a zebrafish model of infection are also in agreement with those results, which provided data showing that hydrophilic *A. baumannii* exhibits increased virulence in *C. elegans* and a murine model of infection, compared to hydrophobic *A. baumannii* strains [32]. Our findings on adhesion and invasion and zebrafish model of infection suggest that Ctp is a virulence determinant and hence can be used as a potential target to develop antimicrobials. The strategies leading to inhibition of the function of *ctp* may be developed for the treatment of *A. baumannii* associated infections. A previous report on alanine-substituted variants of *Tsp* of *E. coli* demonstrated *Tsp* variants with S430A, and K455A substitution lost their catalytic activity [66]. Another report demonstrated the deletion of PDZ domain of *Tsp* also inactivates protease activity [67]. We believe that alanine-substituted variants (S503A and K528A) of *ctp* of *A. baumannii* will also lose their catalytic activity since they have conserved catalytic dyad formed by serine and lysine. Direct relations have been demonstrated between Ctp mutant and virulence using different animal models [34,37], including ours. Studies are in progress to investigate the toxicity of Ctp inactivated strain of *A. baumannii* as per the protocol described previously [68,69]. This study will direct us for further steps that can significantly contribute to the development of novel therapeutics in the future. One of the strategies for the development of therapeutics could be a screening of inhibitors against Ctp of *A. baumannii* that can bind to it and inhibit its activity as reported previously in *E. coli*, when discussing inhibitors against ClpP [70,71].

## 4. Materials and Methods

### 4.1. Bacterial Strains, Plasmids, Primers, and Growth Conditions

The bacterial strains and plasmids used in this study are shown in Table 1. The list of primers used in this study is shown in Table 2. In brief, *A. baumannii* American Type Culture Collection (ATCC) 17978 was used as the wild-type strain. *Escherichia coli* SM10-λ*pir* containing the suicide vector pBSL180 harboring a mini-Tn*10* transposon derivative with a kanamycin resistance cassette (*nptII*) was a generous gift from the National BioResource Project (National Institute of Genetics, Tokyo, Japan). *E. coli* TOP10 (Invitrogen Life Technologies, Carlsbad, CA, USA) was a general cloning host, and we used pTZ57R/T (Fermentas, Waltham, MA, USA) for cloning PCR products according to the manufacturer’s instructions. The pABCL_IIb was used as a shuttle vector between *A. baumannii* and *E. coli. A. baumannii* and *E. coli* were grown in Luria–Bertani (LB) medium at 37 °C and antibiotics were used at the following concentrations: ampicillin (Ap), 100 μg/mL; chloramphenicol (Cm), 30 μg/mL; Apramycin (Apr), 50 μg/mL; and kanamycin (Km), 50 μg/mL.

### 4.2. Mutagenesis of A. baumannii ATCC 17978 by the Mini-Tn10:nptII Transposon Method

For the mutagenesis of *A. baumannii* ATCC 17978, *E. coli* SM10 λ*pir* was used as a donor bacteria harboring the plasmid pBSL180 containing mini_Tn*10*-nptII transposon, and *A. baumannii* as a recipient. For conjugation, a 1:10 volume of donor and recipient were mixed. A nylon membrane (0.45 µm; Bio Trace^TM^ PVDF, Pall Corporation) was placed on the LB agar plate and the mixture of donor and recipient bacteria was placed on the membrane, incubated at 37 °C for 1 h, and resuspended in fresh LB by vortexing. The transconjugants were then spread on an LB plate supplemented with Km and Cm, and incubated overnight at 37 °C. A total of 1500 transconjugants were screened to identify mutants exhibiting defects in motility.

### 4.3. Motility Assay

The transconjugants were screened to get mutants with impaired motility. Following incubation at 37 °C for 16 h, motility was analyzed on 0.5% agar plates supplemented with 5 g/L tryptone and 2.5 g/L NaCl. To reduce variation between plates, 30 mL of medium was poured into each plate in a laminar flow hood and left for 1 h with the lids off, before being used immediately. A 1 µL drop of freshly grown culture (OD_600_ ≅ 0.6) was placed on the center of the plate for surface-associated motility. For twitching motility, the overnight culture was picked with a toothpick and stabbed to the boundary between the bottom of the agar layer and the polystyrene Petri dish (‘interphase’). As a crucial point of the motility assays, the plates were sealed with parafilm to prevent drying and to obtain reproducible results. For each isolate, assays were performed at least three times.

### 4.4. Identification of DNA Sequences at Transposon Insertion Site

The transposon insertion site of the mutants with impaired motility was identified through inverse PCR. Inverse PCR was performed using a thermocycler (GeneAMP^®^ PCR System 2700, Applied Biosystems, Foster City, CA, USA). Total bacterial DNA was purified from mutants by phenol/chloroform extraction, followed by ethanol precipitation. The purified DNA was then digested with the restriction enzyme *Hind*III (Thermo Fisher Scientific Fermentas) at 37 °C for 1 h, followed by self-ligation. *nptII*-specific primers (nptII-iF and nptII-iR, Table 2) were used for the PCR, which employed the following cycling conditions: 95 °C for 5 min, and 35 cycles of 95 °C for 30 s, 52 °C for 30 s, and 72 °C for 1 min, followed by extension at 72 °C for 7 min. The PCR product was then cloned into pTZ57R/T vectors and transformed into *E. coli* TOP 10 cells by spreading on LB agar plates containing Ap. The correct clone was screened by plasmid isolation followed by digestion using restriction sites flanking the insert. The correct clone was then sequenced, and the disrupted gene was identified by NCBI BLAST analysis.

### 4.5. Construction of the A1S_0493 Complementation Strain

The A1S_0493 loci of *A. baumannii* ATCC 17978 were amplified by PCR with the 0493F and 0493R primers which contained a 5’-*Bam*HI and 5’-*Sal*I sites, respectively (Table 2). The resulting 2.317-kb PCR product was ligated into the shuttle vector pABCL_llb to generate pABCL_llb:0493, followed by electroporation into MR14 for complementation.

### 4.6. Purification of eDNA

eDNA was purified from the cell-free supernatant as per the protocol previously described [72]. Briefly, the cell-free supernatants sampled at different time points (up to 24 h) were filtered through 0.22 µm syringe filters. Filtrate (750 µL) was then added to an equal volume of buffer A containing 50 mM Tris-HCl, 10 mM EDTA with 1% cetyl trimethyl ammonium bromide (CTAB), pH 8.0. The solution was incubated for 30 min at 65 °C, centrifuged at 6500× *g* for 10 min, and the pellet was resuspended in 500 µL of buffer B containing 10 mM Tris-HCl, 0.1 mM EDTA, and 1 M NaCl, pH 8.0. To the suspension, 0.3× the volume of ice-cold isopropanol was added, and incubated on ice for 3 h. The solution was centrifuged at 12,000× *g* for 15 min, and the pellet was resuspended in 40 µL of DNase RNase free Tris-EDTA buffer. Proteinase K (10 mg/mL) was added and incubated for 1 h at 37 °C. Finally, it was reprecipitated using ice-cold isopropanol and quantified using the NanoDrop ^TM^ 2000/2000c spectrophotometer.

### 4.7. TEM and SEM Analysis

For the transmission electron microscopy (TEM) analysis of bacteria, bacteria were grown overnight, and 1 mL of the overnight culture was centrifuged at 1000× *g* for 20 min. The supernatant was removed and 1 mL of ice-cold 2.5% glutaraldehyde in 0.1 M cacodylate buffer was used to resuspend the bacterial pellet, before centrifugation at 1000× *g* for 20 min, followed by a final resuspension with 500 µL of ddH_2_O. Ten microliters of the sample was placed on a grid, soaked with filter paper, stained with 10 µL of uranyl acetate, and viewed under a transmission electron microscope (H-7500, Hitachi High-technologies, Tokyo, Japan).

For the scanning electron microscopy (SEM) analysis of bacteria, bacteria were grown overnight; refreshed 1:100 times and incubated at 37 °C until the OD_600_ ≅ 0.6. The culture was diluted to OD ≅ 0.1 in 5 mL of fresh LB, and 1 mL of culture was incubated at 37 °C for 8 h, without shaking, in 24-well plates containing plastic coverslips (13 mm diameter Thermanox^®^). The plastic coverslip was transferred to a new well and washed twice with 1 × PBS, followed by air-drying at 55 °C overnight. Five hundred microliters of 2.5% glutaraldehyde/0.1 M sodium cacodylate buffer was added to the plastic coverslips and incubated on ice (4 °C) for 1 h. The supernatant was removed, and 5% sucrose in 0.1 M sodium cacodylate at 4 °C was used to wash the coverslips for 15 min, before they were stained with 1% of Osmium/0.1 M sodium cacodylate for 1 h at room temperature (21 °C). The coverslips were then washed with 500 µL of 5% sucrose in 0.1 M sodium cacodylate buffer at room temperature for 15 min, before being washed with 50%, 70%, and 95% alcohol each for 10 min, followed by washing with 500 µL of 50% EtOH in 50% HMDS and then 100% HMDS, each for 15 min. The coverslips were dried overnight at 55 °C and viewed with a scanning electron microscope (Hitachi, S-4700, Tokyo, Japan).

### 4.8. Cell Viability Assay

The BacLight^TM^ bacterial viability kit (Invitrogen) containing SYTO9 and PI was used to assess cell viability. The stock solutions of the dyes were prepared as per the manufacturer’s instructions. Briefly, 1 mL of an overnight bacterial culture was centrifuged at 10,000× *g* for 10 min, washed twice and resuspended in 1 × PBS. Four microliters of PI and SYTO9, at working concentrations of 300 and 50 µL/mL, respectively, was added to 100 µL of bacterial solution (OD_600_ ≅ 0.4), and then incubated at 37 °C in the dark. Separate samples were then viewed under the A1^+^ confocal microscope (Nikon) and Axioplan 2, Carl Zeiss Meditech Inc. fluorescence microscope. Additionally, we quantified the number of live and dead bacteria with a ViaQuant^TM^ Viability/Cytotoxicity kit, as per the manufacturer’s instructions.

### 4.9. Stress Tolerance Assays

The stress tolerance test was performed as per the protocol described previously [73]. An overnight culture of bacteria was diluted to an OD_600_ of 0.01 in LB medium, and bacterial growth was measured in the presence or absence of external stress. The stress response to temperature (20 °C), high osmolality (3% NaCl), oxidative stress (20 mM H_2_O_2_), acid (pH 5.5), alkali (pH 8.5), 3% ethanol, and 1% Triton X-100 was measured. The data represents the mean ± SD of three independent experiments.

### 4.10. Cell Surface Hydrophobicity Test

Cell surface hydrophobicity was determined by the bacterial adhesion to hydrocarbons (BATH) test, as per a modified method of that described previously [74]. Briefly, the overnight bacterial culture was centrifuged at 8000× *g* for 5 min, and washed twice with PBS. The cells were then resuspended in PBS, and the OD_600_ was measured (A_1_). Then, 0.5 mL of xylene was added to 3.5 mL of bacterial suspension. The mixture was vortexed vigorously for 20 s, followed by incubation at room temperature for 20 min to allow for separation between the aqueous and solvent phase. The OD_600_ of the aqueous phase was measured (A_2_), and the hydrophobicity was calculated using an equation (A_2_ − A_1_)/A_2_ × 100%.

### 4.11. Adhesion and Invasion Assays

For the adhesion assay, human lung epithelial cells (A549), grown in DMEM supplemented with 10% FBS, were seeded into 24-well cell culture plates and incubated in 5% CO_2_ at 37 °C for 16 h. *A. baumannii* was added to the monolayer at a multiplicity of infection (MOI) of 10 and cultured for 2 h. After washing with PBS thrice, cells were detached with Trypsin/EDTA and lysed with 1% Triton X-100 at 37 °C for 15 min. The diluted lysate was plated onto agar plates and incubated at 37 °C for 16 h. For the invasion assay, the A549 monolayer cells were infected with *A. baumannii* at a MOI of 10 and cultured for 2 h. After washing with PBS, fresh culture medium containing streptomycin (250 µg/mL) was added to each well and incubated for another 2 h. The cells were washed with PBS thrice, and lysed with 1% Triton X-100 at 37 °C for 15 min. The diluted lysate was plated onto agar plates and incubated at 37 °C for 16 h.

### 4.12. Zebrafish Model for Infection

The zebrafish (*Danio rerio*) lines used in the experiments were the wild type AB variety. Mixed male and female populations of zebrafish were kept in 9-L tanks at 28 °C and maintained in a 14 h light/10 h dark cycle. The infection model has been previously reported to study the pathogenesis of *A. baumannii* [75] and showed that *A. baumannii* is lethal to zebrafish in a dose-dependent manner. Here, we first determined the median lethal dose (LD_50_) of *A. baumannii*. For this, the adult disease-free zebrafish were injected with different doses of wild-type strain ATCC 17978 (10^5^, 10^6^, and 10^7^ CFU) via the zebrafish cloaca using an insulin needle after being anesthetized with 0.2% tricaine. Their survival rate was monitored at room temperature by observation every 6 h for 36 h. Further, the zebrafish were divided into three groups (*n* = 14); each group was injected with LD_50_ of ATCC 17978, MR14, and an equal volume of PBS (control group). The fish were transferred into separate tanks following injection, and the survival rate was assessed every 6 h for 24 h. The laboratory protocol was approved by the Institutional Animal Care and Use Committee of Tzu Chi University.

## 5. Conclusions

All data reported here indicate that the *ctp* gene mutation has a pleiotropic effect on the phenotype of *A. baumannii*, affecting motility, cell morphology, stress tolerance, surface hydrophobicity, adhesion and invasion of epithelial cells, and virulence. The pleiotropic phenotype suggests that a large number of proteins are affected by the *Ctp* mutation. The specific target of this gene in *A. baumannii* remains unknown and requires further investigation; however, the experimental data presented here on cell morphology, sensitivity to environmental stress, and bacterial lysis substantiate the role of the *ctp* gene in maintaining cell membrane integrity and stability.

## Figures and Tables

**Table 1:** Bacterial strains and plasmids used in this study.

Strains or Plasmid	Genotype and/or Characteristics	Source
Strains		
*E. coli* SM10λpir	*thiL thrL leuB6 supE44 tonA21 lacY1* for Tn*10* transposon mutagenesis	
ATCC 17978	Wild-type strain	ATCC
MR14	Containing *ctp* gene mutation	This study
MR14C	Containing *ctp* gene complementation	This study
*E. coli* Top10	For the recombinant DNA method	Invitrogen
Plasmids		
pBSL180	Km^r^ Mini-Tn*10* containing vector; R6K *ori* mobRP4	NBRP (NIG, Japan)
pTZ57R/T	Ap^r^ PCR cloning vector	Fermentas
pABCL_llb	Apr^r^ Derived from pET28a, including MCS and N, C-terminal 6XHis tag for complementation	-

ATCC: American type culture collection (Manassas, VA, united states); Km^r^, Kanamycin resistance; Ap^r^, Ampicillin resistance; Apr^r^, Apramycin resistance.

**Table 2:** Primers used in this study.

Primers	Sequence (5’-3’)	DNA Template	Restriction Site
FP-nptII	AGATGGATTGCACGCAGG	pBSL180	-
RP-nptII	CCACAGTCGATGAATCCAGA	Tn*10* transposon	-
*npt*II-iF	GCCTTCTATCGCCTTCTTGAG	pBSL180	-
*npt*II-iR	TACGTGTTCCGCTTCCTTTAG	pBSL180	-
0493F	GGATCCCACCCAAGGAAGTATTAAG	ATCC 17978	*BamH*I
0493R	GTCGACTGACACTTTGATGATAAATGC	ATCC 17978	*Sal*I
FP-0493	GGATCCGCTGTTTCTCAATCGATCC	ATCC 17978	*BamH*I
RP-0493	GTCGACGTTCTGCAATTTCGCATA	ATCC 17978	*Sal*I

Underline: Primers.
